# Validation of Computer Vision for Segmenting Timed Up and Go Subtasks from Conventional Video Recordings

**DOI:** 10.1093/geroni/igaf122.3867

**Published:** 2025-12-31

**Authors:** Chitra Banarjee, Sarah Reynolds, Zengyan Wang, Chen Chen, Rui Xie, Ladda Thiamwong

**Affiliations:** University of Central Florida, Orlando, Florida, United States; University of Central Florida, Orlando, Florida, United States; University of Central Florida, Orlando, Florida, United States; University of Central Florida, Orlando, Florida, United States; University of Central Florida, Orlando, Florida, United States; University of Central Florida, Orlando, Florida, United States

## Abstract

The Timed Up and Go (TUG) test is a standardized clinical tool used to assess the dynamic balance of older adults for fall prevention. It is typically assessed using a stopwatch recording the total duration to complete the test. Recently, studies have focused on the relevance of the subtasks of the TUG: sit-to-stand, 3-meter forward-walk, turn, back-walk, stand-to-sit. Studies using wearable devices or depth cameras have introduced new metrics for assessing fall risk in clinical settings. These metrics have been shown to be associated with lower limb strength, motor impairments, and executive function. We aimed to utilize an affordable video camera and computer vision (CV) to detect the durations of TUG components and validate it through comparison with manual coding. The sample included 17 older adults (70.6 + 7.3 years, 70.6% female), who completed four trials of the TUG. The trials were recorded on a GoPro Hero 12 video camera, placed 110 inches away from the turning point with a frontal view of the participant. The videos were segmented into subtasks by trained research assistants and analyzed using AlphaPose and MotionBERT for 2D and 3D human pose estimation, respectively. Subtasks were extracted using the head and shoulder coordinates from the CV output. Spearman correlations were utilized to compare the annotated and CV-extracted durations of the subtasks, revealing comparable durations (ρ = 0.479, p < 0.001). These preliminary results indicate the potential of CV in primary care settings as an affordable method of expanding clinical evaluation of dynamic balance in older adults by medical professionals.

